# Pectin modulates intestinal immunity in a pig model via regulating the gut microbiota-derived tryptophan metabolite-AhR-IL22 pathway

**DOI:** 10.1186/s40104-023-00838-z

**Published:** 2023-03-08

**Authors:** Guoqi Dang, Xiaobin Wen, Ruqing Zhong, Weida Wu, Shanlong Tang, Chong Li, Bao Yi, Liang Chen, Hongfu Zhang, Martine Schroyen

**Affiliations:** 1 State Key Laboratory of Animal Nutrition, Institute of Animal Sciences, Chinese Academy of Agricultural Sciences, Beijing, 100193 China; 2 Precision Livestock and Nutrition Unit, Gembloux Agro-Bio Tech, TERRA Teaching and Research Centre, Liège University, Passage des Déportés 2, Gembloux, Belgium; 3 The Key Laboratory of Feed Biotechnology of Ministry of Agriculture, National Engineering Research Center of Biological Feed, Feed Research Institute, Chinese Academy of Agricultural Sciences, No.12 Zhongguancun South Street, Haidian District, Beijing, 100081 China

**Keywords:** Dietary fiber, Gut microbiota, Immune pectin, Tryptophan metabolites

## Abstract

**Background:**

Pectin is a heteropolysaccharide that acts as an intestinal immunomodulator, promoting intestinal development and regulating intestinal flora in the gut. However, the relevant mechanisms remain obscure. In this study, pigs were fed a corn-soybean meal-based diet supplemented with either 5% microcrystalline cellulose (MCC) or 5% pectin for 3 weeks, to investigate the metabolites and anti-inflammatory properties of the jejunum.

**Result:**

The results showed that dietary pectin supplementation improved intestinal integrity (Claudin-1, Occludin) and inflammatory response [interleukin (*IL)-10*], and the expression of proinflammatory cytokines (*IL-1β*, *IL-6*, *IL-8, TNF-α*) was down-regulated in the jejunum. Moreover, pectin supplementation altered the jejunal microbiome and tryptophan-related metabolites in piglets. Pectin specifically increased the abundance of *Lactococcus*, *Enterococcus*, and the microbiota-derived metabolites (skatole (ST), 3-indoleacetic acid (IAA), 3-indolepropionic acid (IPA), 5-hydroxyindole-3-acetic acid (HIAA), and tryptamine (Tpm)), which activated the aryl hydrocarbon receptor (AhR) pathway. *AhR* activation modulates *IL-22* and its downstream pathways. Correlation analysis revealed the potential relationship between metabolites and intestinal morphology, intestinal gene expression, and cytokine levels.

**Conclusion:**

In conclusion, these results indicated that pectin inhibits the inflammatory response by enhancing the AhR-IL22-signal transducer and activator of transcription 3 signaling pathway, which is activated through tryptophan metabolites.

## Introduction

Weaning is one of the most critical periods in both animal production and infant growth and development. The gastrointestinal tract of animals is not fully developed at this stage [1]. It is susceptible to changes in feeding patterns and nutrition, leading to stress and diarrhea. Given the omnivorous and physiological similarities between weaned piglets and human infants, the piglet is regarded as the most suitable animal model for studying gut health [2].

Pectin, predominantly composed of α-1,4-linked *D*-galacturonic acid (GalA) monomers, is abundant in citrus, apple, lemon peels and pulp. As a typical fermentable dietary fiber, pectin can regulate the human and animal intestinal microbiota [3–5]. It can also strengthen the mucus layer to restrict the entry of hazardous substances [6, 7]. Furthermore, it can enhance the integrity of the epithelial cell layer [8] and maintain intestinal integrity in piglets exposed to lipopolysaccharide or high-fat diet [9].

The gastrointestinal tract is home to a diverse community of trillions of microorganisms collectively known as the gut microbiota [10], and this intricate community is central to gut health and disease [11]. Moreover, the gut microbiota is associated with its ability to defend against enteropathogens, absorb nutrients, and maintain a healthy immune system [12–14]. However, pectin can also have direct effects in the small-intestinal sites [7]. It has been shown that the non-esterified GalA residues rich in pectin can bind to toll like receptor 2 (TLR2) via ionic bonds [15]. The pectin suppresses the TLR2/1 signal (TLR2 can form heterodimers with TLR1), and then IL-6 secretion is reduced, thereby reducing the inflammatory response and ameliorating the damage [16].

Recently, many studies have focused on the function of microbial tryptophan catabolites in the gut and their contributions to host physiology [17]. For instance, aryl hydrocarbon receptor (AhR) ligands; 3-indole ethanol (IE), 3-indole pyruvate (IPA), and 3-indole aldehyde (IA) reduce gut permeability [18]. Serotonin (5-HT), another catabolite, plays an important role in gastrointestinal absorption, transit, and secretion. Besides, it also regulates mood, behavior, pain modulation, and cognitive function via the central nervous system [19]. According to a representative study, pectin supplementation may not only alter the intestinal flora of mice, but also increase the tryptophan metabolites of the flora by activating the AHR pathway [20]. Previous research from our laboratory has demonstrated the anti-inflammatory effects of pectin on gut immunity [21]. However, the precise mechanism by which pectin promotes gut health remains unknown.

Thus, the intriguing question was whether microbial tryptophan catabolite is the link between pectin and the gut health regulator. To bridge this knowledge, we examined the changes in serum, gut microbiota, and tryptophan metabolite following pectin supplementation in al pig model. This study gave a novel perspective for promoting a new understanding of how pectin promotes gut health.

## Materials and methods

### Ethics statement

All animal experiments were approved by the Animal Ethics Committees of Institute of Animal Sciences, Chinese Academy of Agricultural Sciences (Ethics Code Permit IAS2019-37).

### Standards and chemicals

Pectin extracted from citrus peel (Henan Yuzhong Biotechnology Co., Ltd., Zhengzhou, China) mainly consisted of galacturonic acid (white powder, with purity of > 81.4%, DM: 13.5%). Microcrystalline cellulose (MCC) is a β-1,4-multi-bonded linear carbohydrate consisting of glucose residues with 99.5% purity. (Beijing NCC Technology R&D Center, China). Reference standards for tryptophan (Trp), tryptamine (Tpm), 3-indoleacetic acid (IAA) and kynurenine (Kyn) were purchased from Sigma-Aldrich (St. Louis, MO, USA); 5-hydroxyindole-3-acetic acid (HIAA) and skatole (ST) were obtained from Cato Research Chemicals Inc. (Eugene, OR, USA); 3-indolylpropionic acid (IPA) and serotonin (5-HT) were from Laboratory of the Government Chemist (Teddington, UK) and Beijing Wokai Biotechnology Co., Ltd. (China), respectively. Assay kits, including interleukin IL-17, IL-22 were purchased from Nanjing Jiancheng Bioengineering Institute (Jiangsu, China).

### Experimental design and animal care

A total of 16 crossbred barrows aged 21 d (6.77 ± 0.92 kg; Duroc × Landrace × Yorkshire) were randomly assigned to one of two diets with eight piglets per treatment. No antibiotics were administered to the piglets throughout the 4-week experiment. Piglets were fed ad libitum and had free access to water. A corn-soybean basal diet was formulated to meet nutritional requirements of National Research Council (NRC, 2012) [22]. After a 3-d of adaption, piglets were fed a diet containing either 5% microcrystalline cellulose (w/w) as the control (CON) group or 5% pectin (w/w) as the treatment (PEC) group for 3 weeks. All piglets were housed in separated pens with daily-cleaned plastic slatted floors.

### Sample collection

Blood samples were acquired from the jugular vein via a sterilized syringe before the pigs were sacrificed at the end of the experiment. The serum was then separated by centrifugation for 10 min at 3000 × *g* at 4 °C and stored in aliquots at −80 °C for cytokines analysis. The middle section (2 cm) of the jejunum was obtained and fixed in 4% paraformaldehyde for histological examination. The intestinal segment was washed with ice-cold phosphate buffered saline (PBS), and the mucosa was scraped off using a glass microscope slide. Mucosa samples were immediately snap-frozen in liquid nitrogen and stored at −80 °C to further investigate the bacterial community, genes, and protein expression.

### Intestinal morphology

The hematoxylin-eosin (HE) staining of the jejunum was performed according to the methods as previously described [23]. Briefly, specimens of jejunum were embedded in paraffin, sectioned (5 μm thickness), and stained with HE for histological evaluation [villus height (VH)]. Microphotographs were taken with a Leica DM2000 light microscope (Leica, Wetzlar, Germany) at a magnification of 40. VH was performed using Image Pro software [24].

### Serum inflammatory cytokines

The ELISA kit was employed to detect serum cytokines as previous describe [25]. Quantitative analysis of pro-inflammatory cytokine (IL-17), and anti-inflammatory cytokine (IL-22) in serum were measured by ELISA kits (Nanjing Jiancheng Bioengineering Institute, Nanjing, China) according to the detection kit instructions.

### Quantitative real-time (qRT) PCR analysis

Total RNA was extracted from the jejunum mucosa, using the RNeasy Mini Kit (GeneBetter, Beijing, China). The concentration of each RNA sample was quantified using the NanoDrop 2000 (Nanodrop Technologies, Wilmington, DE, USA). The cDNA was transcribed at 37 °C for 15 min and 85 °C for 5 s using the PrimeScriptTM RT reagent kit with gDNA Eraser (Takara Biomedical Technology in Beijing, China). qRT-PCR with 40 amplification cycles was conducted with a commercial kit (PerfectStart Green qPCR SuperMix, TransGen Biotech, Beijing, China). In detail, a total of 10 μL reaction mixture contain 1 μL of cDNA, 0.4 μL forward primer, 0.4 μL reverse primer, 0.2 μL of ROX, and 3 μL of PCR-grade water. The gene of β-actin was used as an internal control. Primers used were listed in Table 1. The relative gene expression level between the control group and the treatment group was calculated by the 2^-ΔΔCt^ method, and the value was normalized to the internal control.

**Table 1:** Primer sequences used for real-time PCR

Gene	Primer	Nucleotide sequences (5′ to 3′)
β-actin	F	GCGTAGCATTTGCTGCATGA
	R	GCGTGTGTGTAACTAGGGGT
*ZO-1*	F	CTCCAGGCCCTTACCTTTCG
	R	GGGGTAGGGGTCCTTCCTAT
Occludin	F	CAGGTGCACCCTCCAGATTG
	R	TATGTCGTTGCTGGGTGCAT
Claudin-1	F	TCGACTCCTTGCTGAATCTG
	R	TTACCATACCTTGCTGTGGC
*IL-1β*	F	GCCAGTCTTCATTGTTCAGGTTT
	R	CCAAGGTCCAGGTTTTGGGT
*IL-6*	F	TCCAATCTGGGTTCAATCA
	R	TCTTTCCCTTTTGCCTCA
*IL-8*	F	TACGCATTCCACACCTTTC
	R	GGCAGACCTCTTTTCCATT
*IL-10*	F	TCGGCCCAGTGAAGAGTTTC
	R	GGAGTTCACGTGCTCCTTGA
*IL-17*	F	CTCTCGTGAAGGCGGGAATC
	R	GTAATCTGAGGGCCGTCTGG
*TNF-α*	F	CGTCGCCCACGTTGTAGCCAAT
	R	GCCCATCTGTCGGCACCACC
*AhR*	F	CATGCTTTGGTCTTTTATGC
	R	TTCCCTTTCTTTTTCTGTCC
*CYP1A1*	F	CCTTCACCATCCCTCACAGT
	R	ATCACCTTTTCACCCAGTGC
*CYP1B1*	F	AATAACGGGGGAAATTCCTG
	R	CACCGAAACACAATGCAATC
*RegIIIγ*	F	AACCTGGATGGGTGCAGACGTG
	R	TTGGTTCCAAGCCCTCGGTG
*IL-22*	F	CTACATCACCAACCGCACCT
	R	TCAGAGTTGGGGAACAGCAC

*ZO-1* Zonula occludens-1, *IL-1* Interleukin 1, *IL-6* Interleukin 6, *IL-8* Interleukin 8, *IL-10* Interleukin 10, *IL-17* Interleukin17, *TNF-α* Tumor necrosis factor-alpha, *CYP1A1* Cytochrome P450, family 1, subfamily A, polypeptide 1, *CYP1B1* Cytochrome P450, family 1, subfamily B, polypeptide 1, *IL-22* Interleukin 22

### Western blotting assay

Total protein was extracted from jejunum tissue using RIPA lysis buffer (Thermo Fisher Scientific Inc., Waltham, MA, USA). It was quantified with the BCA protein assay kit (Cat# 23225, Thermo, Waltham, MA, USA). Total proteins in the amount of 30 μg were loaded for separation onto 10% SDS-PAGE. The proteins were then transferred onto a polyvinylidene difluoride (PVDF) membrane at 90 V for 1.5 h using the wet transfer method. The membranes were then incubated in 5% skimmed milk for 2 h at room temperature for blocking. After incubation with a primary antibody Occludin (Thermo Fisher Scientific Inc., #40-4700, 1:5000), Claudin-1 (Thermo Fisher Scientific Inc., #51-9000, 1:5000), IL-22 (Abcam, #ab193813, 1:2000), STAT3 (Biowordtechnology; #AP0365, 1:1000), P-STAT3 (Biowordtechnology; #AP0248, 1:1000), and β-actin (CST, #4970 T, 1:4000) overnight at 4 °C, the membranes were incubated with HRP-labeled goat anti-mouse or goat anti-rabbit secondary antibodies (1:5000). Protein blots were visualized using a gel imaging system (Tanon 2500R; Tanon Science & Technology Co., Ltd., Shanghai, China). The band density was quantified using Image J 10.0 software and normalized to β-actin.

### 16S ribosomal RNA (rRNA) amplicon sequencing

Genomic DNA was extracted from the jejunum mucosa using the EZNA^TM^ Soil DNA Kit (D5625-02, Omega Bio-Tek Inc., Norcross, GA, USA), as directed by the manufacturer. The hypervariable region V3-V4 of the bacterial 16S rRNA gene was amplified by a two-step PCR using specific primers (338F, 5′-ACTCCTACGGGAGGCAGCAG-3′ and 806R, 5′-GGACTACH VGGGTWTCTAAT-3′) with unique 8-bp barcodes to facilitate multiplexing. The amplicons were sequenced using the Illumina HiSeq sequencing platform, as previously described. The Majorbio Cloud Platform (www.majorbio.com) was used to analyze the raw data. The raw reads were deposited to the Sequence Read Archive (SRA) database (Accession Number: PRJNA876628) of NCBI. A more detailed methodology was described previously [21].

### Trp and its metabolites analysis by liquid chromatograph-mass spectrometer (LC-MS)

Methanol was used to extract Trp and its metabolites (ST, IAA, IPA, HIAA, Tpm, 5-HT, Kyn) from the jejunum mucosa. The methanol extraction solutions were pre-cooled for 30 min at −20 °C. After being vortexed for 1 min, the samples were grinded 3 times (30 s for each time and 10 s intervals) with high throughput Tissuelyser instrument (Scientz-48, Jingxin, Shanghai, China). The supernatant was collected after centrifugation at 10,000 × *g* for 5 min and filtered through 0.22 μm filter membranes (Jin Teng, Tianjin, China).

LC-MS analysis was performed on Agilent 1290 UHPLC electrospray ionization-time-of-flight mass spectrometer (ESI-TOF-MS) coupled with Agilent 1260 SFC-Ultivo equipped with an Agilent ZORBAX Eclipse XDB-C18 column (3.0 mm × 150 mm, 1.8 μm). A linear gradient was obtained by mixing eluent A (water + 0.1% formic acid) and eluent B (100% methanol). The elution gradient for 5-HT and ST was as follows: 0–0.5 min (20% B), 0.5–1 min (20%–40% B), 1–3 min (65%–75% B), 3–4 min (75%–90% B), 4–7 min (90%–100% B) at the flow rate of 0.5 mL/min. For the remaining metabolites (Trp, IAA, IPA, HIAA, Tpm, Kyn), the elution gradient was set as follows: 0–0.5 min (20% B), 0.5–1 min (20%–40% B), 1–2 min (40%–50% B), 2–3 min (50%–80% B), 3–4 min (80% B), 4–7 min (80%–85% B), 7–9 min (85%–100% B), 9–11 min (100% B), and the flow rate was 0.3 mL/min, and the column temperature was 40 °C. The amount of each metabolite was calculated according to standard curves with known metabolite levels.

### Statistical analysis

Data conforming to normal distribution were compared using Student *t*-test, while those with non-normally distributed were tested using Kruskal-Wallis test (*CYP1A1*, serum IL-17, *TNF-α*). These analyses were performed using the JMP software (JMP R version 10.0.0, SAS Institute, Cary, NC, USA) for Windows.

Raw data obtained from gut microbiota were processed using the free online platform of Majorbio I-Sanger Cloud Platform (www.i-sanger.com). For β-diversity, principal-coordinate analysis (PCoA) plots were produced using Bray-Curtis distances, and community significance was confirmed with a Wilcoxon Rank-Sum test. All data were presented as mean ± standard error of the mean (SEM). Acceptable significance levels were at ^∗^*P* < 0.05 and ^∗∗^*P* ≤ 0.01.

Spearman’s or Mantel’s correlation was used to analyze the correlation between the mucosal tryptophan metabolites, gene expression (inflammatory cytokines, STAT3/IL-22 pathway), and tryptophan and its derivatives in the jejunum.

## Results

### Dietary pectin supplementation improved the integrity of jejunum

**Fig. 1:**
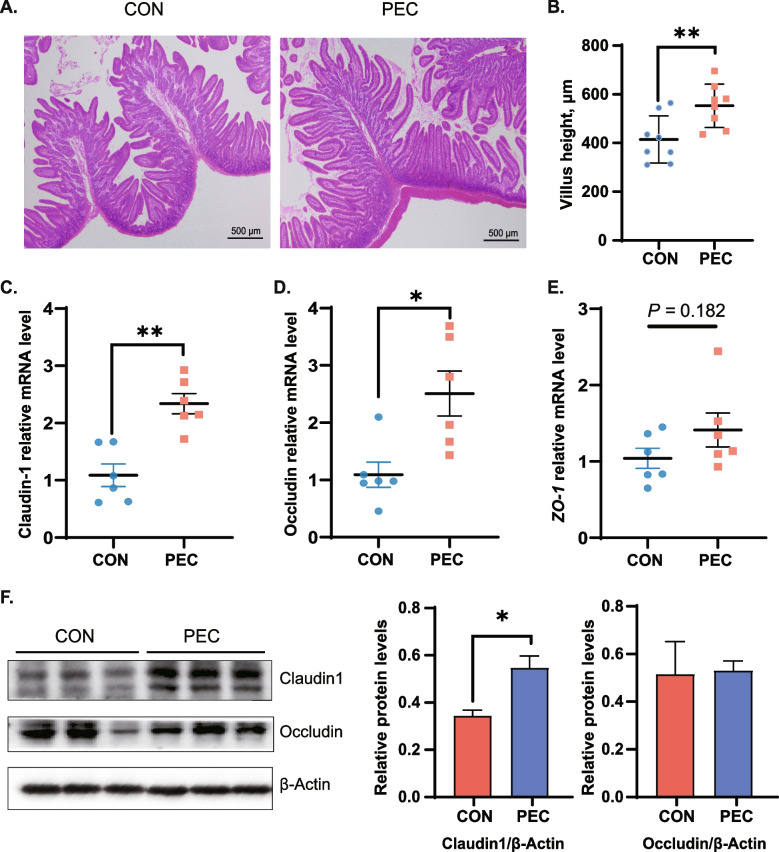
Effects of pectin on jejunal morphology in piglets. **A** Representative images of hematoxylin-eosin staining in the jejunum; **B** Jejunal villus height; **C** Jejunal mRNA expression levels of Claudin-1; **D** Jejunal mRNA expression levels of Occludin; **E** Jejunal mRNA expression levels of *ZO-1* (*n* = 6). **F** Jejunal protein expression levels of tight junction proteins (Occludin, Claudin-1) (*n* = 4). Data were expressed as mean ± SEM. ^*^P < 0.05, ^**^P < 0.01, ^***^P < 0.001

To determine the effects of pectin supplementation on intestinal integrity, HE staining, qPCR, and western blotting methods were used to examine the jejunum morphology and tight junctions. Histopathology staining results showed that the villus height was increased significantly (*P* < 0.05) in the PEC group than in control (Fig. 1A–B). Additionally, the mRNA expression levels of tight junction proteins Claudin-1 (*P* = 0.005*)*, Occludin (*P* = 0.016), and zonula occludens-1 (*ZO-1, P* = 0.108) were increased (Fig. 1C–E). Western blotting results showed that the protein level of Claudin-1 increased greatly (*P* < 0.05), however, the level of Occludin did not change significantly (Fig. 1F). It was indicated that pectin supplementation improved intestinal barrier and gut integrity.

### Pectin supplementation altered the expression levels of inflammatory cytokines in the jejunal mucosa and serum

**Fig. 2:**
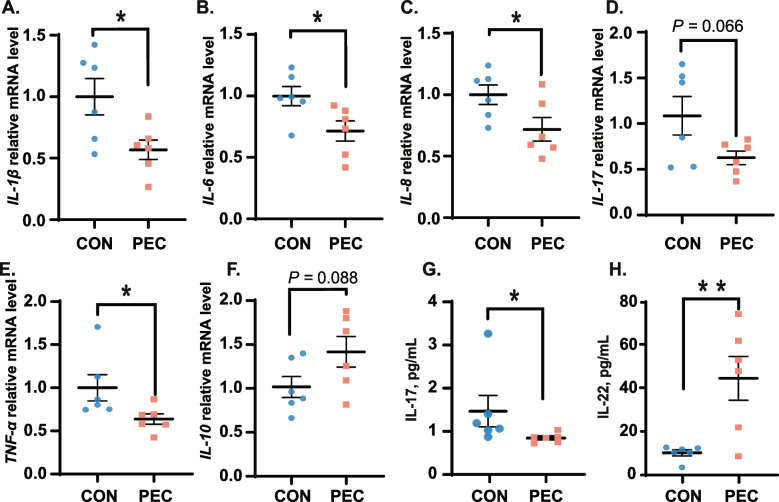
Pectin supplement altered the expression levels of inflammatory cytokines in the jejunum and serum. **A ***IL-1β*; **B ***IL-6*; **C ***IL-8*; **D ***IL-17*; **E ***TNF-α*; **F ***IL-10*; **A**–**F** were detected levels in Jejunum; **G ***IL-17*; and **H ***IL-22* were detected in serum, *n* = 6. ^*^P < 0.05; ^**^P < 0.01; ^***^P < 0.001; data are presented as the mean ± SEM (*n* = 6)

The inflammatory cytokines were also detected in the jejunal mucosa and serum. In the jejunal mucosa, pectin supplementation downregulated the expression of pro-inflammatory cytokines, *IL-1β* (Fig. 2A; *P <* 0.05), *IL-6* (Fig. 2B; *P <* 0.05), *IL-8* (Fig. 2C; *P <* 0.05), *IL-17* (Fig. 2D; *P =* 0.066), and *TNF-α* (Fig. 2E; *P <* 0.05). On the other hand, the expression of the anti-inflammatory cytokine *IL-10* (Fig. 2F; *P =* 0.088) was increased in the PEC group compared to the control group. Additionally, after pectin supplementation, a diminished expression level of IL-17 (Fig. 2G; *P <* 0.05) and an enhanced expression level of IL-22 (Fig. 2H; *P* < 0.008) was observed in the serum. Thus, pectin supplementation in the diet regulated the jejunum inflammatory responses in piglets.

### Pectin supplementation altered the bacterial community in jejunum mucosa

Following size filtering, quality control, and chimera checking, 16S rRNA amplicon sequencing results revealed a total of 859,243 reads ranging from 35,227 to 74,138 reads per sample, to examine the effect of pectin on microbial population in the jejunum. Sequencing counts were normalized to acquire normalized reads for each sample into operational taxonomic units (OTUs) based on 97% identity.

**Fig. 3:**
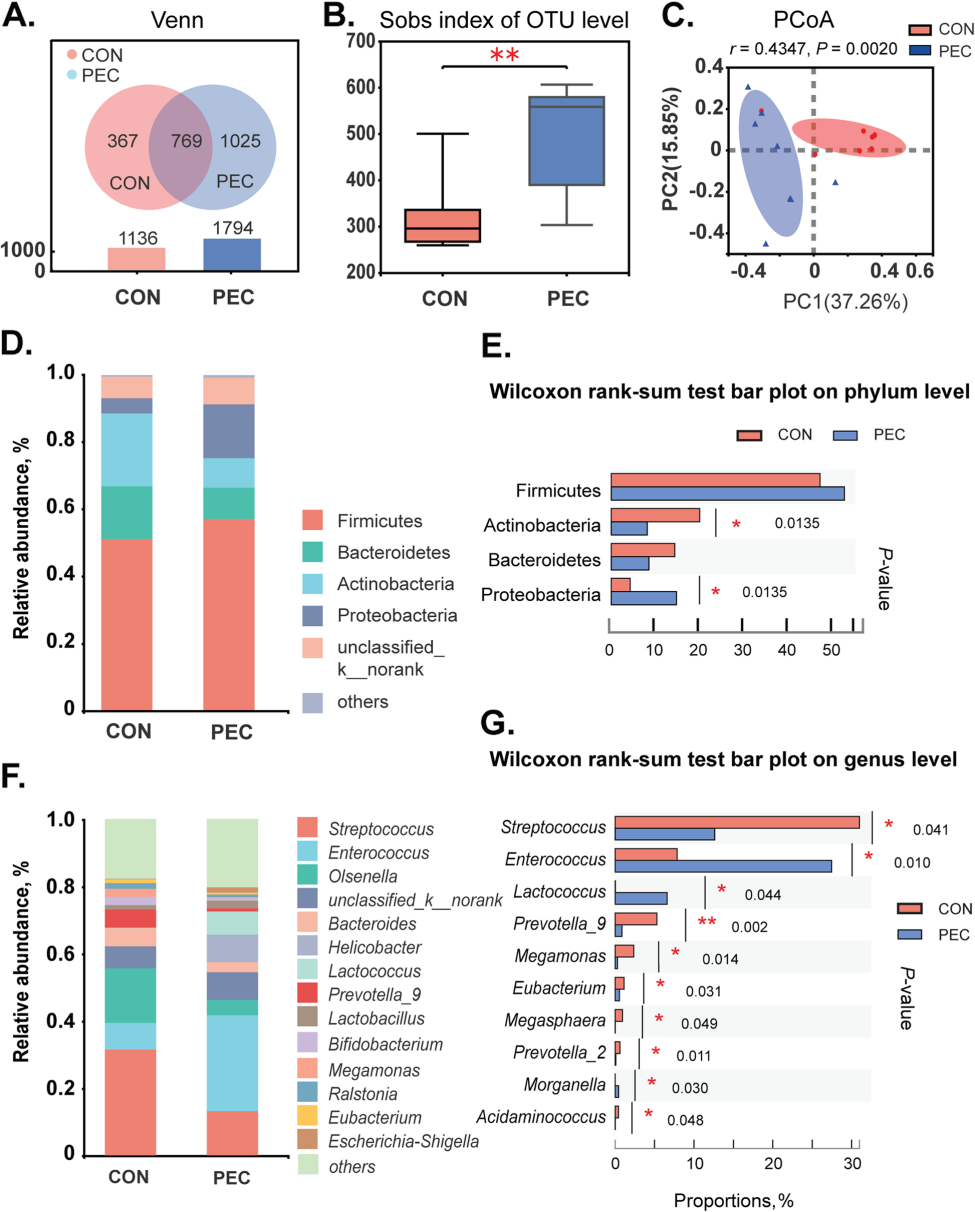
Effects of pectin on the jejunum microbial diversity. **A** Venn diagram; **B** The alpha diversity indices (Sobs); **C** The beta diversity presented by the PCoA plot based on the OTU level; **D** The abundance of the intestinal microbiota composition at the phylum level; **E** Differences in microbial community composition between two groups at phylum level; **F** The abundance of the intestinal microbiota composition at the genus level; **G** Differences in microbial community composition between two groups at phylum level. Data were expressed as mean ± SEM, *n* = 8. ^*^P < 0.05; ^**^P < 0.01; ^***^P < 0.001

As indicated in Fig. 3, a Venn diagram was utilized to reveal the common and unique OTUs in the control and/or pectin supplementation groups. Pigs in the CON and pectin groups had 367 and 1025 distinct OTUs, respectively, and 769 common OTUs (Fig. 3A). Additionally, alpha diversity (Sobs indexes) revealed that the gut microbial flora diversity of pectin-treated piglets was significantly different from that of the control piglets, at the OTU level in the jejunal mucosa (Fig. 3B). This was further supported by the beta diversity presented in PCoA (Fig. 3C). The composition of the gut microbial community was then analyzed at the genus level. Firmicutes, Bacteroidetes, Actinobacteria, and Proteobacteria constituted the majority of the microbiota at the phylum level (Fig. 3D). Pectin boosted the abundance of Proteobacteria, whereas decreased the abundance of Actinobacteria (Fig. 3E). Noticeable alterations in their microbial composition were detected at the genus level (Fig. 3F). Pectin significantly reduced the relative abundance of *Streptococcus*, *Prevotella_9*, *Megamonas*, *Eubacterium*, *Megasphaera*, *Prevotella_2*, and *Actidaminococcus*, whereas it increased the relative abundance of *Enterococcus*, *Lactococcus*, and *Morganella* (Fig. 3G, *P* < 0.05).

### Pectin altered the levels of microbiota-derived tryptophan metabolites in jejunum

**Fig. 4:**
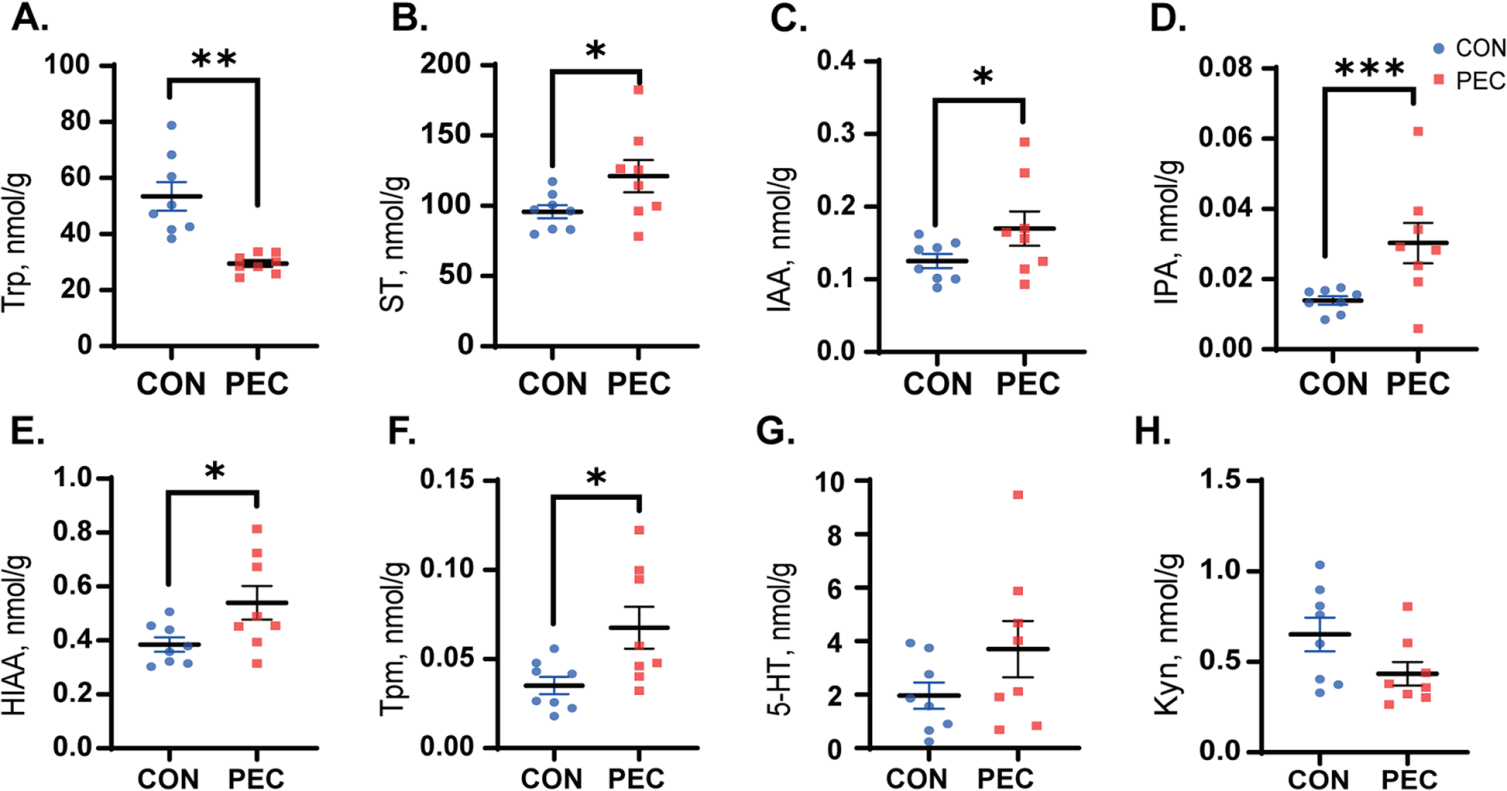
Effect of pectin on the jejunum microbial tryptophan metabolism concentration. **A** Trp (tryptophan); **B** ST (Skatole); **C** IAA (3-Indole acetic acid); **D** IPA (3-indolepropionic). **E** 5-Hydroxyindole-3-acetic acid; **F** Tpm (Tryptamine); **G** 5-HT (5-hydroxytryptamine); **H** Kyn (Kynurenine). Data are presented as the mean ± SE, (*n* = 8). ^*^P < 0.05; ^**^P < 0.01; ^***^P < 0.001

Trp is an important metabolite related to gut microbiota. Various diets and bacterial populations influenced the concentration of Trp and its derivatives. The Trp-derived metabolites in the jejunal mucosa were determined to evaluate whether a change in the intestinal microbiota affects the production of Trp and its related metabolites after pectin supplementation. The concentration of Trp was significantly lower in the pectin group compared to the CON group (Fig. 4A). As for indole derivatives, the concentrations of ST (Fig. 4B), IAA (Fig. 4C), 3- IPA (Fig. 4D), HIAA (Fig. 4E), and Tpm (Fig. 4F) were significantly higher in the pectin-fed piglets than in the CON group (*P <* 0.05). Particularly, the content of IPA reached extremely significant levels (Fig. 4D; *P <* 0.001). Additionally, two other pathway metabolites, 5-HT and Kyn were not significantly different between these two groups (Fig. 4G–H). Accordingly, adding pectin facilitated tryptophan metabolism towards the indoles as AhR ligands in the piglet intestine.

### The changed Trp metabolism by pectin supplementation activated the AhR/IL-22/STAT3 signaling pathway in jejunal mucosa of piglets

**Fig. 5:**
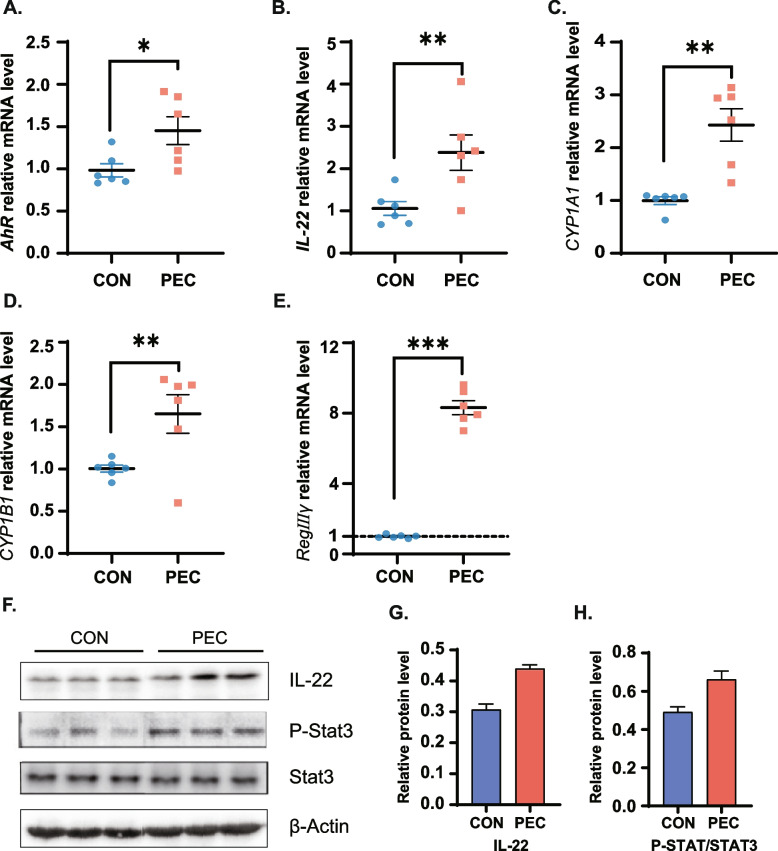
Dietary pectin supplementation influenced *AhR* activation and relative downstream genes expression in the jejunum of weaned piglets. **A ***AhR*; **B ***IL-22*; **C ***CYP1A1*. **D ***CYP1B1*. **E ***RegIIIγ*. **F** Protein abundances of IL-22, STAT3 and p-STAT3; **G** Protein abundances of IL-22; **H** The protein rate of p-STAT3/STAT3. Data are presented as the mean ± SE, (*n* = 6). ^*^P < 0.05; ^**^P < 0.01; ^***^P < 0.001

Microbial derived tryptophan catabolites (indole compounds) are ligands for the AhR and act on the AhR in lymphoid cells. Therefore, we investigated the effect of pectin on the AhR signaling pathway. We analyzed the expression of *AhR* activation in the jejunum. All changes in expression [*AhR* (Fig. 5A), *IL-22* (Fig. 5B), cytochrome P450 1A1 (*CYP1A1*, Fig. 5C), cytochrome P450 1B1 (*CYP1B1*, Fig. 5D), recombinant regenerating islet derived protein 3 gamma (*RegIIIγ*, Fig. 5E)] were significantly increased (*P* < 0.05). Similar results were obtained using WB analysis of the IL-22-STAT3 pathway (Fig. 5F). The protein levels of IL-22 and P-STAT/STAT3 were increased, however, not to a significant level (Fig. 5G–H, *P* > 0.05). These results mentioned above suggested that pectin can activate the AhR-IL-22-STAT3 signaling pathways.

### AhR activation in mucosa is potentially correlated with mucosal tryptophan metabolites

**Fig. 6:**
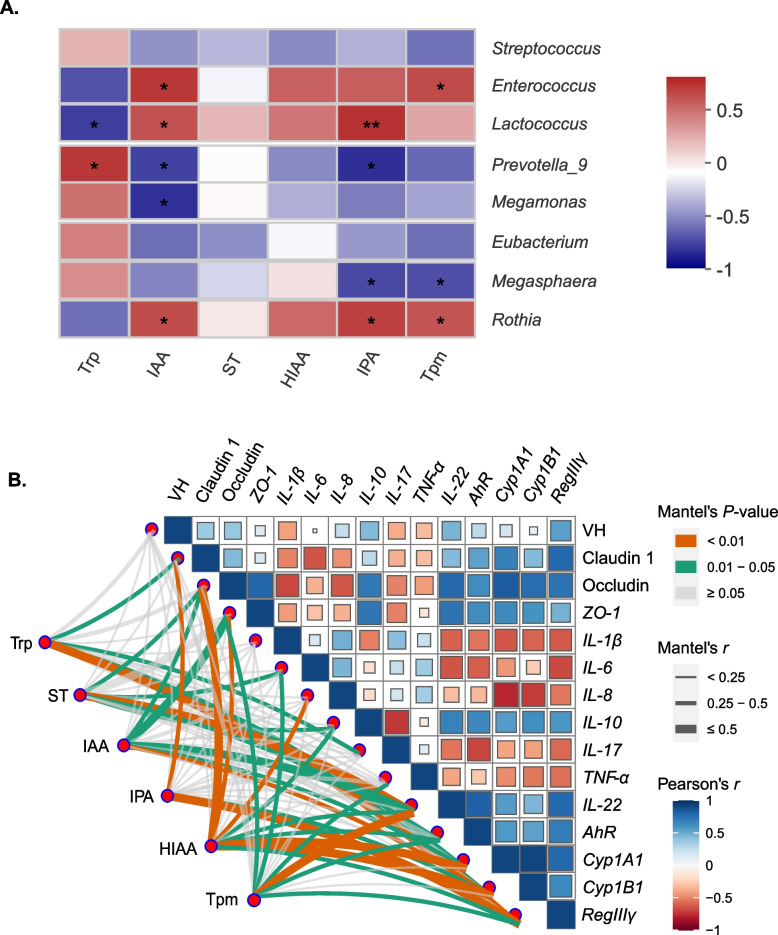
Heat maps of the Spearman rank correlation coefficient and significant tests between the differential bacteria and tryptophan metabolites (**A**). Pairwise comparisons if metabolites are demonstrated with a color gradient denoting Spearman’s correlation coefficient. Trp, ST, IAA, IPA, HIAA, and Tpm are related to inflammatory cytokines, jejunum morphology indices by partial spearman tests. Edge width corresponds to the Partial Spearman’s *r* statistic for the corresponding distance correlations and edge color denotes the statistical significance (**B**). Data are presented as the mean ± SE, (*n* = 6). ^*^P < 0.05; ^**^P < 0.01; ^***^P < 0.001

Spearman rank correlations coefficient and significance tests revealed a correlation between the various bacteria and the tryptophan metabolites (Fig. 6A). The concentration of Trp was significantly and negatively correlated with the abundance of *Lactococcus*, whereas it was significantly and positively linked to the abundance of *Prevotella_9*. The concentration of IAA was positively correlated with the abundance of *Enterococcus*, *Lactococcus*, and *Rothia*, but inversely correlated with *Prevotella_9* and *Megamonas*. IPA was positively correlated with the abundance of *Lactococcus* and *Rothia*, while it was negatively correlated with *Prevotella_9* and *Megasphaera*. Tpm had a significantly positive relationship with *Enterococcus* and *Rothia*, whereas it had a significantly negative relationship with *Megasphaera*.

Moreover, the Mantel test demonstrated a significant correlation between Trp and the gene expression levels of Claudin-1, *IL-17*, *CYP1A1*, and *RegIIIγ* (Mantel’s *r* > 0.25, *P* < 0.05, Fig. 6B). Beyond that, five genes (Occludin*, IL-6, IL-10, IL-22, AhR*) showed a significant correlation with ST (Mantel’s *r >* 0.25, *P <* 0.05). IAA significantly correlated with Occludin*, ZO-1*, *IL-22*, *CYP1A1*, and *CYP1B1*. Additionally, we found that Claudin-1, *CYP1B1*, and *RegIIIγ* showed a significant association with IPA. HIAA was also associated with eight genes (Occludin*, ZO-1, IL-10, IL-22, AhR, CYP1A1*, *CYP1B1,* and *RegIIIγ*). There was a significant relationship between Tpm and *ZO-1, IL-6, TNF-α, IL-22, AhR*, and *RegIIIγ*.

## Discussion

During the weaning transition period, piglets are susceptible to infection by various pathogens and non-pathogens, associated with a disrupted state of microbiota and an immature immune system [26]. Emerging evidence demonstrated that pectin could enhance anti-inflammatory properties, regulate the host microbiome [27], boost markers of mucus barrier function, modulate immunological activity [21, 28], and promote gut integrity [29]. In this study, we demonstrated that supplementing piglets with pectin can boost their anti-inflammatory activity, which may be associated with changes in microbial tryptophan metabolites induced by pectin supplementation.

In this study, pectin was found to increase the villus height, suggesting that it enhances intestinal health status and promotes nutrient absorption. Previous research has also shown that dietary fiber treatment can improve the morphological structure of the jejunum of piglets, as evidenced by an increase in VH and a decrease in crypts depth (CD), resulting in decreased intestinal mucosal permeability [24, 30] and increased the intestinal barrier protection [31, 32].

As an essential protein in the intestine, tight junction proteins play a crucial role in gut barrier function, particularly in maintaining the integrity of the intestinal barrier and preventing the spread of harmful substances [33]. Recent research showed that feeding piglets inulin or pectin increases the gene expression of Claudin-1, Occludin, and *ZO-1* [21, 34]. As expected, the results of our study were consistent with the previous work. Thus, pectin supplementation during the weaning transition period improved the intestinal barrier function of piglets.

Subsequently, we also investigated whether pectin may have an additional beneficial effect on the intestinal tract. As is well-known, *TNF-α*, *IL-1β*, and *IL-6* are essential proinflammatory response indicators. Additionally, macrophages produce *IL-8*, a small inflammatory cytokine. This indicates that variations in these proinflammatory cytokine levels may reflect the inflammatory response status. A previous study revealed that low-methoxyl pectin might downregulate the mRNA levels of *TNF-α*, *IL-1β*, and *IL-6* in ileum colonic tissues [35]. Furthermore, pectin extract from apples might decrease the gene expression of *IL-6* in mouse ileum tissue [9]. Similarly, pectin derived from oranges and lemons can improve intestinal inflammation by inhibiting the initiation of *IL-6* secretion [15]. In this study, we observed that administration of pectin reduced the mRNA expression of *IL-1β*, *IL-6*, *IL-8*, and *TNF-α* in jejunal mucosa of piglets. Moreover, there are several trials suggested that a low degree of methyl-esterification-(DM) pectin could suppress TLR2-TLR1 by directly blocking of TLR2 receptor [36], then slow down capsule implantation induced increasing in pro-inflammatory cytokine (*TNF-α*, *IL-6*), and promoted the release of *IL-10* [37]. *IL-17* is also a proinflammatory cytokine mainly produced by Th17 cells and is associated with the pathogenesis of many autoimmune inflammatory diseases [38]. *IL-10,* which is primarily secreted by Tregs, reduces Th17 development and function, as well as inhibits the secretion of proinflammatory cytokines and chemokines [39]. Research showed that the decreased expression of *IL-10* and the increased expression of *IL-17* might aggravate intestinal inflammation [40]. Interestingly, the pectin treatment decreased the mRNA expression level of *IL-17* while increasing the mRNA expression level of *IL-22*, which was consistent with the data mentioned above from the previous studies. Moreover, the levels of serum cytokines IL-17 and IL-22 were consistent with those of the jejunal mucosa. Thus, we proposed that pectin may modulate mucosal immunity by increasing anti-inflammatory cytokines and decreasing pro-inflammatory cytokines.

Due to its fermentation properties on microorganisms, most previous studies on dietary fiber focused on the hindgut [41, 42]. In contrast, pectin was found to modulate the microbial composition of the foregut in this study, although the foregut is not the primary site of microbial fermentation. Wu et al. [21] reported that the supplementation of pectin in the piglet diet decreased the diversity and abundance of microorganisms in the small intestine. Similar results were obtained in other studies [43]. In contrast, other studies observed an increased abundance and diversity of ileal microbial in piglets [44, 45]. We also hypothesized that pectin supplementation could affect gut health by altering the microbial composition or in other ways. As predicted by the preceding analysis, our data revealed that pectin significantly changed the composition and structure of the gut microbiota and increased the OTU number and alpha diversity. A healthy gut microbiota typically consists of four main phyla: Firmicutes, Bacteroidetes, Actinobacteria, and Proteobacteria [46]. In the present study, we observed that pectin reduced the abundance of Actinobacteria in the jejunal mucosa. It has been reported that the abundance of Actinobacteria was highly expressed in the gut of goats with diarrhea [47]. Proteobacteria, an intestinal commensal bacteria, was also significantly increased by pectin in the present study. However, pectin significantly decreased the abundance of *Streptococcus*, a known facultative-anaerobe bacterium and an opportunistic pathogen. Specifically, *Streptococcus* infection may cause mucosal damage [48] and is associated with an increased risk of colorectal cancer [49]. Our study also found that the indole-derivatives-producing bacteria (*Lactobacillus* and *Enterococcus*) in the gut showed a notable increase in pectin group. Additionally, pectin significantly reduced the abundance of *Prevotella_9*, *Megamonas*, *Eubacterium*, *Megasphaera*, *Prevotella_2*, and *Acidaminococcus*. *Prevotella_9* and *Megasphaera* are generally regarded as opportunistic pathogens [50, 51]. Moreover, *Megamonas*, *Eubacterium*, and *Acidaminococcus* are commonly believed to be associated with fatty acid metabolism [52–55], which significantly decreased in pectin group. *Morganella*, the Gram-negative bacillus, belongs to the Enterobacteriaceae family. This study observed that pectin supplementation significantly increased the abundance of *Morganella*. Therefore, our study showed that adding pectin significantly reduced the abundance of harmful bacteria (*Streptococcus*) and increased the abundance of beneficial bacteria (*Enterococcus* and *Lactobacillus*) in the intestinal mucosa, to promote intestinal health.

*Lactobacillus* and *Enterococcus* are related to tryptophan metabolism. Specifically, tryptophan is an important amino acid that mammals must obtain from their diet, and it can be transformed into indole and indole derivatives by the gut microbiota [56, 57]. Then, it can alter the immune-related signaling pathways that regulate inflammation in the gut [58]. Only a few commensal species, including *Enterococcus* [59, 60] and *Lactobacillus* [61], are known to produce indole derivatives, and many more are likely to be discovered. In this study, pectin supplementation decreased the concentration of Trp while increasing the amount of indole derivatives (ST, IAA, IPA, HIAA, Tpm). Previous studies suggested that dietary fiber supplementation increased the levels of indole derivates, including IPA [62]. Other studies also showed that pectin can alleviate alcoholic hepatitis by promoting the elevation of microbial metabolites, IAA, and Indole-3-lactic acid [20]. This accorded with our research that pectin promotes the metabolism of tryptophan, shifting the metabolic direction toward the metabolism of indoles. As a whole, pectin increased the content of indole derivatives in the gut, which had a beneficial effect on intestinal immunity.

Group 3 innate lymphoid cells (ILC3) are greatly enriched in the lamina propria of the jejunum, the highly expressed *AhR* in ILC3 can be activated by tryptophan metabolites (indole derivates) as ligands, thereby promoting the production of IL-22 by ILC3 cells [63–66], and activates downstream pathways by inducing phosphorylation of Stat3, further promoting the production of antimicrobial peptides and mucins [67, 68]. Representative research showed that pectin supplementation altered the intestinal flora of mice, increased the tryptophan metabolites of the flora, and reduced alcohol-induced liver damage by activating the AhR pathway [20, 29]. In this study, we found that pectin treatment enhanced the levels of *AhR*, *IL-22* and *p*-*Stat3* downstream of *AhR*, which plays as an essential role in promoting the production of antimicrobial molecules (*CYP1A1*, *CYP1B1*, and *RegIIIγ*). Consequently, these antimicrobial molecules exerted the protective function of the intestinal barrier. This indicated that the altered in the microbiota structure and metabolite concentration in jejunal mucosa observed following pectin treatment was the basis of immunomodulation, with the activated AhR/IL-22/Stat3 signaling pathway providing a plausible mechanism.

## Conclusions

In conclusion, our study found that adding pectin to the model could improve the intestinal integrity and gut immunity by promoting tryptophan metabolism. This indicated that dietary pectin supplementation altered jejunal microbial composition, thus promoting microbial tryptophan metabolism. Increased metabolites can act as ligands or signaling molecules to modulate the intestinal immune response through the AhR/IL-22/State3 pathway, ultimately reducing proinflammatory factors and enhancing intestinal barrier function. These results provided a reliable theoretical foundation and guidance for using pectin in mammals as a prospective intestinal health defender.

## Data Availability

The datasets used and/or analyzed during the current study are available from the corresponding author on reasonable request.

## Abbreviations

AhR: Aryl hydrocarbon receptor
CYP1A1: Cytochrome P450 1A1
CYP1B1: Cytochrome P450 1B1
GalA: D-galacturonic acid
HE: Ematoxylin-eosin
HIAA: 5-Hydroxyindole-3-acetic acid
IA: Indoleacrylic acid
IAA: 3-indoleacetic acid
IAALD: 3-indole acetaldehyde
IALD: 3-indole aldehyde
IE: 3-indole ethanol
IL: Interleukin
IPA: 3-indolepropionic acid
Kyn: Kynurenine
MCC: Microcrystalline cellulose
PVDF: Polyvinylidene difluoride
SRA: Sequence Read Archive
ST: Skatole
TPm: Tryptamine
Trp: Tryptophan
VH: Villus height
ZO-1: Zonula Occludens-1
5-HT: Serotonin
